# Using the National Death Index to Identify Duplicate Cancer Incident Cases in Florida and New York, 1996–2005

**DOI:** 10.5888/pcd11.140200

**Published:** 2014-09-25

**Authors:** Brad Wohler, Baozhen Qiao, Hannah K. Weir, Jill A. MacKinnon, Maria J. Schymura

**Affiliations:** Author Affiliations: Brad Wohler, Jill A. MacKinnon, Florida Cancer Data System, Miami, Florida; Baozhen Qiao, Maria J. Schymura, New York State Cancer Registry, Albany, New York.

## Abstract

**Introduction:**

Cancer registries link incidence data to state death certificates to update vital status and identify missing cases; they also link these data to the National Death Index (NDI) to update vital status among patients who leave the state after their diagnosis. This study explored the use of information from NDI linkages to identify potential duplicate cancer cases registered in both Florida and New York.

**Methods:**

The Florida Cancer Data System (FCDS) and the New York State Cancer Registry (NYSCR) linked incidence data with state and NDI death records from 1996 through 2005. Information for patients whose death occurred in the reciprocal state (the death state) was exchanged. Potential duplicate cases were those that had the same diagnosis and the same or similar diagnosis date.

**Results:**

NDI identified 4,657 FCDS cancer patients who died in New York and 2,740 NYSCR cancer patients who died in Florida. Matching identified 5,030 cases registered in both states; 508 were death certificate-only (DCO) cases in the death state’s registry, and 3,760 (74.8%) were potential duplicates. Among FCDS and NYSCR patients who died and were registered in the registry of the reciprocal state, more than 50% were registered with the same cancer diagnosis, and approximately 80% had similar diagnosis dates (within 1 year).

**Conclusion:**

NDI identified DCO cases in the death state’s cancer registry and a large proportion of potential duplicate cases. Standards are needed for assigning primary residence when multiple registries report the same case. The registry initiating the NDI linkage should consider sharing relevant information with death state registries so that these registries can remove erroneous DCO cases from their databases.

## MEDSCAPE CME

Medscape, LLC is pleased to provide online continuing medical education (CME) for this journal article, allowing clinicians the opportunity to earn CME credit.

This activity has been planned and implemented in accordance with the Essential Areas and policies of the Accreditation Council for Continuing Medical Education through the joint sponsorship of Medscape, LLC and Preventing Chronic Disease. Medscape, LLC is accredited by the ACCME to provide continuing medical education for physicians.

Medscape, LLC designates this Journal-based CME activity for a maximum of 1 **AMA PRA Category 1 Credit(s)™**. Physicians should claim only the credit commensurate with the extent of their participation in the activity.

All other clinicians completing this activity will be issued a certificate of participation. To participate in this journal CME activity: (1) review the learning objectives and author disclosures; (2) study the education content; (3) take the post-test with a 75% minimum passing score and complete the evaluation at www.medscape.org/journal/pcd (4) view/print certificate.

**Release date: September 25, 2014; Expiration date: September 25, 2015**

### Learning Objectives

Upon completion of this activity, participants will be able to:

- Describe death certificate-only cases in the death state’s cancer registry identified as duplicates using the National Death Index, and potential duplicate cases, based on a study using the Florida Cancer Data System and New York State Cancer Registry.
- Determine possible reasons why the National Death Index identified deaths among state residents that were missed through routine death clearance.
- Discuss potential strategies to avoid duplicate cases in state cancer registries.

**EDITORS**

Ellen Taratus, Editor, *Preventing Chronic Disease*. Disclosure: Ellen Taratus has disclosed no relevant financial relationships.

**CME AUTHOR**

Laurie Barclay, MD, Freelance writer and reviewer, Medscape, LLDisclosure: Laurie Barclay, MD, has disclosed no relevant financial relationships.

**AUTHORS AND CREDENTIALS**

Disclosures: Brad Wohler, MS; Baozhen Qiao, PhD; Hannah K. Weir, PhD; Jill MacKinnon, PhD; Maria J. Schymura, PhD have disclosed no relevant financial relationships.

Affiliations: Hannah Weir, Division of Cancer Prevention and Control, Centers for Disease Control and Prevention, Atlanta, Georgia; Brad Wohler, Jill MacKinnon, Florida Cancer Data System, Miami, Florida; Baozhen Qiao, Maria J. Schymura, New York State Cancer Registry, Albany, New York.

## Introduction

In the United States, there is a population-based cancer registry in all 50 states, Puerto Rico, the US Pacific Island jurisdictions, the District of Columbia, and 6 metropolitan areas funded by the Centers for Disease Control and Prevention’s (CDC’s) National Program of Cancer Registries (NPCR) and/or the National Cancer Institute’s (NCI’s) Surveillance, Epidemiology, and End Results (SEER) Program. Registries collect information on all residents newly diagnosed with or treated for cancer in their catchment areas and report de-identified data to their federal surveillance programs and to the North American Association of Central Cancer Registries (NAACCR), where the data are combined for the purpose of reporting national, regional, and state cancer incidence data (1–4).

Cancer registries collect data using procedures and codes promulgated by NAACCR (5), including personal and demographic data (eg, name, social security number, birth date, race/ethnicity, sex, residence at diagnosis) and case data (eg, date of diagnosis, primary site, histology, behavior). Cancer patients can be diagnosed with multiple primary cancers; therefore, each cancer case is assigned a sequence number according to the temporal order in which it was diagnosed. Registries also collect follow-up information, including data on vital status (alive or dead), date of last contact, and cause of death.

Deaths are recorded by the vital records office in the state where the death occurred, and if applicable, the death certificate is forwarded, per data-exchange agreement, to the vital records office in the state where the person resided at the time of death. Each year, cancer registries perform death clearance (6), the process by which registries link their incidence data with their state death certificates to identify cancer patients who may have been missed by the registry at the time of their diagnosis and to update vital status (deaths) among registered cancer patients. If no information is found in the registry for a cancer diagnosis on these death certificate–initiated cases, they are registered as death certificate–only (DCO) cases in the cancer registry’s database.

However, patients may move out of state between the time of their diagnosis and their death. When an incident record does not match with a state death certificate record, the cancer registry may send patient personal and demographic data for linkage with CDC’s National Death Index (NDI) (7). The NDI is a repository of death certificate information from vital statistics offices in all 50 states, the District of Columbia, and Puerto Rico. To encourage NDI linkages, CDC and NCI have arranged for NDI linkage services to be available at no cost to the registries.

An accurate count of incident cases is necessary for understanding the burden of cancer in the population and for planning health resources to meet the growing cancer burden. However, the data that are reported to the federal surveillance programs do not contain personal identifiers and, therefore, it is not possible to identify duplicate cases — incident cases that have the same diagnosis and similar diagnosis dates and are reported by 2 or more cancer registries. Duplicate case reporting can lead to over-counting incident cases and create an inaccurate picture of the cancer burden at the state and national levels.

The Florida Cancer Data System (FCDS) and the New York State Cancer Registry (NYSCR) routinely perform death clearance with their state death certificate records, and both have sent personal and demographic data on incident cases to NDI. The objective of this study was to explore the use of information from NDI linkages to identify potential duplicate cancer cases registered in both the FCDS and the NYSCR.

## Methods

### Source of data

The FCDS began as a population-based cancer registry in 1981 and received funding from NPCR for the reporting of incidence data beginning in 1995. The FCDS registers approximately 100,000 new incident cases per year. The NYSCR has been population-based since 1976 and received funding from NPCR for the reporting of incidence data beginning in 1996. The NYSCR registers approximately 100,000 new incident cases per year. Both registries conduct annual death clearance and in 2008, both registries sent data to NDI for linkage to information on deaths that occurred through December 31, 2005.

Per agreement with NCHS, the registry that initiates the NDI linkage may share NDI-derived information (except for cause of death) with the cancer registry in the state where the death occurred (death state) to determine whether the cancer patient was registered in that state’s cancer registry. Both FCDS and NYSCR have agreements that allow for the exchange of data between the 2 registries.

Common procedures were used to prepare data for linkage with NDI and process linkage results (8). FCDS staff identified deaths in New York among cancer patients registered in the FCDS; conversely, NYSCR staff identified deaths in Florida among cancer patients registered in the NYSCR. Exchange files were prepared by each registry; the files included personal and demographic information (name, birth date, social security number, sex, and race/ethnicity), case information (diagnosis date, primary site, histology, behavior, laterality, sequence number, and type of reporting source), and data from the NDI linkages (death date, death certificate number). Each record included a 5-digit SEER site recode, which was assigned on the basis of information on primary site and histology (9). When a patient had multiple cancer diagnoses, information on all cases was included in the exchange files.

### Data linkage and analysis

We examined linkage between the FCDS and NYSCR and their state death certificate records and the NDI for deaths from 1996 through 2005 among cancer cases diagnosed during the same period. Exchange files were matched to the cancer registry database by using algorithms developed by the registry. If a match was found, further comparisons were made, including whether the incident case was registered as a single primary (ie, only) cancer or one of multiple primary cancers, whether the case was registered as a DCO case in the death state registry, and whether the SEER site codes matched. If the match was not a DCO case, and if the first 4 digits of the SEER site recode were the same, the diagnosis dates were compared for Florida residents who died in New York and were registered in the NYSCR and for New York residents who died in Florida and were registered in the FCDS.

## Results

In Florida, 997,290 cancer cases were diagnosed among Florida residents and registered in the FCDS (Table 1). During the same period, 434,526 deaths were reported among these cancer patients. Linkage with state death certificate records identified 398,196 (91.6%) deaths, all of which occurred in Florida, and linkage with NDI identified 36,330 (8.4%) additional deaths. Among NDI-identified deaths, 3,464 occurred in Florida and 32,866 occurred outside of Florida; 4,657 of these occurred in New York.

**Table 1:** Linkage of State Vital Records and National Death Index (NDI) for Cancer Patients in the Florida Cancer Data System (FCDS) and the New York State Cancer Registry (NYSCR), 1996–2005

Category	FCDS, n	NYSCR, n
**Cancer incident cases diagnosed**	997,290	952,663
**Deaths identified among incident cases**	434,526	401,463
**Deaths identified through linkage with state death certificate records**	398,196	389,164
Died in state where diagnosis was made	398,196	383,343
Died outside of state where diagnosis was made	0	5,821
Died in reciprocal state^a^	0	1,488
**Deaths identified through linkage with NDI**	36,330	12,299
Died in state where diagnosis was made	3,464	1,398
Died outside of state where diagnosis was made	32,866	10,901
Died in reciprocal state^a^	4,657	2,740
Died in another state	28,209	8,161

a The reciprocal state for Florida is New York, and the reciprocal state for New York is Florida.

In New York, 952,663 cancer cases were diagnosed among New York residents and registered in the NYSCR (Table 1). During the same period, 401,463 deaths were reported among these patients. Linkage with state death certificate records identified 389,164 (96.9%) deaths; of these, 383,343 occurred in New York and 5,821 occurred outside of the state; approximately 26% occurred in Florida. Linkage with NDI identified 12,299 (3.1%) additional deaths. Among NDI-identified deaths, 1,398 occurred in New York and 10,901 deaths occurred outside of New York; 2,740 (25%) deaths occurred in Florida.

Of the 2,740 patients who were registered in the NYSCR and died in Florida, 66.0% (1,808/2,740) matched to a patient in the FCDS database (Table 2). Among matched patients, 15.2% (275/1,808) were registered as a single primary DCO case and 69.0% (1,248/1,808) were registered with at least 1 record indicating the same diagnosis site reported by the NYSCR.

**Table 2:** Match Results Between Exchange Files and Death State Cancer Registries for Deaths of Cancer Patients in the Florida Cancer Data System (FCDS) and the New York State Cancer Registry (NYSCR), 1996–2005

Category	NYSCR, n	FCDS, n
**Exchange files received from reciprocal state for linkage^a^**	4,657	2,740
**Matched to a patient in registry of reciprocal state^b^**	3,222	1,808
Registered as a single primary DCO^c^ incident case	233	275
Registered as non-DCO^c^ incident case	2,989	1,533
At least 1 record has same first 4 digits of the SEER site recode	2,512	1,248
First 4 digits of SEER site recode are different in all records	477	285

Abbreviations: SEER, Surveillance, Epidemiology, and End Results.

a The National Death Index identified 4,657 FCDS cancer patients who died in New York and 2,740 NYSCR cancer patients who died in Florida.

b The reciprocal state for Florida is New York, and the reciprocal state for New York is Florida.

c When a state cancer registry obtains information on the death of a cancer patient through a death certificate, but no information is found in the state registry for a cancer diagnosis, these death certificate–initiated cases are registered as death certificate–only (DCO) cases in the cancer registry’s database.

Of the 4,657 patients who were registered in the FCDS and died in New York, 69.2% (3,222/4,657) matched to a patient in the NYSCR database (Table 2). Among matched patients, 7.2% (233/3,222) were registered as a single primary DCO case and 78.0% (2,512/3,222) were registered with at least 1 record indicating the same diagnosis site reported by FCDS.

Of the 3,760 patients registered in both the FCDS and NYSCR, 4,035 potential duplicate cancer cases were identified: 948 (23.5%) lung and bronchus, 472 (11.7%) colorectal, 373 (9.2%) female breast cancer, 358 (8.9%) prostate cancer, 266 (6.7%) urinary bladder, and 1,618 (40.1%) other cancers (Table 3). Among matched cases, the difference between the diagnosis dates was 6 months or less for 73.7% of cases, 1 year or less for 81.7% of cases, and more than 2 years for 12.3% of cases; and 72.2% of all matched cases were reported as a single primary case in the cancer registry.

**Table 3:** Matched-Case Results Among Cancer Patients Who Were Registered In Both the Florida Cancer Data System (FCDS) and the New York State Cancer Registry (NYSCR) With One or More Similar Diagnoses, 1996–2005^a^

Category	NYSCR and FCDS	NYSCR	FCDS
**Total patients, n**	3,760	2,512	1,248
**Total non-DCO^b^ incident cases that matched, n**	4,035	2,673	1,362
**Matched Cases**			
**Cancer site**			
Lung and bronchus	948 (23.5)	686 (25.7)	262 (19.2)
Colorectal	472 (11.7)	296 (11.1)	176 (12.9)
Female breast	373 (9.2)	223 (8.3)	150 (11.0)
Prostate	358 (8.9)	227 (8.5)	131 (9.6)
Urinary bladder	266 (6.7)	165 (6.2)	101 (7.4)
All others	1,618 (40.1)	1,076 (40.2)	542 (39.8)
**Difference in time (in months) between diagnosis dates in records of matched cases**			
≤6	2,970 (73.7)	2,017 (75.5)	953 (70.0)
7–12	321 (8.0)	199 (7.4)	122 (9.0)
13–24	246 (6.1)	156 (5.8)	90 (6.6)
≥25	498 (12.3)	301 (11.3)	197 (14.5)
**Sequence number^c^**			
Single primary	2,912 (72.2)	2,043 (76.4)	869 (63.8)
First primary of multiple primaries	582 (14.2)	306 (11.5)	276 (20.3)
Second or later primary of multiple primaries	541 (13.4)	324 (12.1)	217 (15.9)

a All values are number (percentage) unless otherwise indicated.

b When a state cancer registry obtains information on the death of a cancer patient through a death certificate, but no information is found in the state registry for a cancer diagnosis, these death certificate–initiated cases are registered as death certificate–only (DCO) cases in the cancer registry’s database.

c When cancer patients are diagnosed with multiple primary cancers, each cancer case is assigned a sequence number according to the temporal order in which it was diagnosed.

## Discussion

This study provides evidence that cancer patients were registered in both the FCDS and the NYSCR, and that linkage with the NDI identified DCO cases that could be removed from the registry in the state where the death occurred. NDI linkages also identified potential duplicate cancer cases. Procedures are needed for determining residence at diagnosis for cancer patients registered in more than one cancer registry with the same or related diagnosis.

As in previous findings (10), linkage with state death certificates identified the majority of deaths among deceased cancer patients registered in the FCDS (91.6%) and the NYSCR (96.9%), whereas linkage with the NDI identified additional deaths among patients who died in the state where they were registered and deaths that occurred in a different state.

NDI identified deaths that were missed through routine death clearance for several possible reasons. First, death clearance is usually performed once per death year and near the end of the diagnosis year. If an incident record or death certificate is reported late to the registry or vital records offices, the record or certificate may not be available at the time of death clearance. Second, the matching algorithms used by the registries differ from those used by NDI; the former uses probabilistic linkages while the latter uses multiple deterministic linkages (11), and deaths may be missed by one or the other. However, a side-by-side comparison of state (probabilistic) and NDI (deterministic) linkage results using Georgia incidence and death data yielded similar results (K. C. Ward, PhD, MPH, Georgia Center for Cancer Statistics, personal communication), so this difference in algorithms probably does not explain the missing deaths. Third, some data-exchange agreements, such as those with the Florida vital records office, may limit the use of exchanged death certificate information to statistical reporting. As a result, the FCDS was missing information on nearly 1% of deceased cancer patients who died out of state and whose death certificates were not shared with the FCDS. Fourth, death clearance is used to ascertain missing incident cases, and registry staff may use only cancer-related deaths to expedite the process. If a patient did not die from cancer, the death would be missed.

In this study, most NDI matches were for deaths that occurred among cancer patients who were diagnosed in one state (the diagnosis state) and moved and died as a resident of a different state (the death state). Because the death occurred to a resident of a different state, the cancer registry in the diagnosis state would have no knowledge of the death if the registry relies on linkage with state death certificate records to ascertain deaths.

Most cancer patients who died in a different state than the one in which they were presumably diagnosed were also registered as incident cases in the death state’s cancer registry. Among the 4,657 FCDS patients who died in New York State, 69.2% were registered in the NYSCR and of the 2,740 NYSCR patients who died in Florida, 66.0% were registered in the FCDS. A small number of these patients (233 in the NYSCR and 275 in the FCDS) were reported as DCO cases in the death state’s cancer registry. These cases could be removed from the registry regardless of their cause of death because the cancer was diagnosed while the patient was a resident in a different state. Death certificates do not always accurately reflect the true cause of death (12,13); therefore, it seems reasonable to remove these cases regardless of agreement between the coded cause of death and the coded cancer at diagnosis. 

Recently, the FCDS and the NYSCR sent data on incident cases spanning their complete years of operations to NDI and conducted data exchange with a focus on removing erroneous DCO cases. Through this process, 1,300 additional DCO cases were removed from the NYSCR database (B.Q., unpublished data, 2012). However, this process left numerous potential duplicate cases that were registered as non-DCO incident cases. Of these, most were registered in the other state’s cancer registry with the same diagnosis in both registries. Most of these cases were reported as a single primary cancer in both registries and had similar diagnosis dates. Further investigation is needed to resolve these potential duplicate case reports. To resolve these cases, it will be necessary to develop procedures to determine usual residence at diagnosis (5). For example, a cancer patient may reside in more than one state during the year, spending winter months in a southern state, such as Florida, and summer months in a northern state, such as New York. For the purpose of being enumerated by the US Census, only one usual residence is recognized (14). If similar rules were adopted by cancer registries, it might help ensure agreement between the numerator (incident cases) and the denominator (population counts) when calculating cancer incidence rates.

Information on patients who are diagnosed or treated for cancer, regardless of where they reside, is required by public law (15) to be reported to the cancer registry in the state where the medical services are performed. The registry then sends this information to the cancer registry in the state where the patient resides, provided the patient’s correct residence is known and data-exchange agreements are in place. This scenario is complicated when a cancer patient changes residence in the middle of care, for example, to be closer to family and friends. Without querying the cancer patient directly, it may be difficult to identify the original place of residence.

The issue of duplicate case reporting within cancer registries is well recognized (16,17). NAACCR has standards for reporting high-quality cancer incidence data, which include the requirement that registries have one or fewer duplicate cases per 1,000 cases reported (18). However, the issue of duplicate case reporting between and among registries has, to our knowledge, not been raised.

Cancer registries in Australia (19), Canada (20), Sweden (21), and the United Kingdom (22) routinely report personal identifiers as well as demographic and case data to their national cancer surveillance organizations. For example, the Canadian Cancer Registry (CCR) is a patient-based system maintained by Statistics Canada (20). The 13 provincial and territorial cancer registries annually report data to the CCR, which conducts internal record linkages to detect duplicate records and match incidence records to death records in its national mortality database (23). Conflicting information and duplicate case reports are resolved through consultation with the provincial and territorial cancer registries that reported the information. In addition, the CCR identifies multiple primary cancers in a cancer patient when the first primary cancer is diagnosed in one province or territory and the subsequent primary cancer is diagnosed in another.

Unlike Canada and elsewhere, the 2 US cancer surveillance systems (NPCR and SEER) are primarily case-based systems. Because NPCR and SEER registries do not report personal identifiers to their surveillance programs, identifying inter-registry duplicate case reports or multiple primary cancer cases registered in the same patient but in different cancer registries is not possible.

Although the total number of potential duplicate cases identified in this study was less than 1% of all incident cases in both the FCDS and the NYSCR, the finding that most deceased cancer patients whose deaths were reported in both the FCDS and the NYSCR had the same cancer site (and of these, a similar date of diagnosis) suggests that the impact of duplicate case reporting could be much higher if linkages were extended to include living cancer patients and were performed among cancer registries in the United States. Many US registries operate in small geographic areas and in population centers close to bordering states (Figure). The challenges posed by inter-registry duplicate cases among cancer registries in the United States are likely to increase: the number of annual cancer incident cases is projected to double from 2000 to 2050 (24); the US population is mobile (25); and cancer patients are living longer after diagnosis (2,26).

**Figure:**
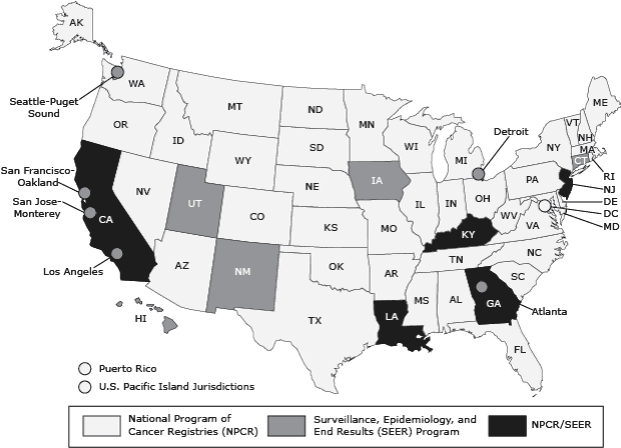
State, territory, and metropolitan-area cancer registries participating in the National Program of Cancer Registries or the Surveillance, Epidemiology, and End Results programs or both programs as of 2000. Source: US Cancer Statistics Working Group (27).

NDI identified DCO cases that were, but should not have been, registered in the death state’s cancer registry. Registries initiating NDI linkages should consider sharing relevant information with death state registries to remove erroneous DCO cases in the death state’s registry. NDI also identified a large proportion of potential duplicate cases. Standards are needed to assign primary residence when multiple registries report the same case. This study probably underestimated the number of potential duplicate cases between the FCDS and the NYSCR because the analysis was limited to deceased patients.

## Figures and Tables

**Table:** 

**1. The activity supported the learning objectives.**				
**Strongly Disagree**				**Strongly Agree**
1	2	3	4	5
**2. The material was organized clearly for learning to occur.**				
**Strongly Disagree**				**Strongly Agree**
1	2	3	4	5
**3. The content learned from this activity will impact my practice.**				
**Strongly Disagree**				**Strongly Agree**
1	2	3	4	5
**4. The activity was presented objectively and free of commercial bias.**				
**Strongly Disagree**				**Strongly Agree**
1	2	3	4	5

## Post-Test Information

To obtain credit, you should first read the journal article. After reading the article, you should be able to answer the following, related, multiple-choice questions. To complete the questions (with a minimum 75% passing score) and earn continuing medical education (CME) credit, please go to http://www.medscape.org/journal/pcd. Credit cannot be obtained for tests completed on paper, although you may use the worksheet below to keep a record of your answers. You must be a registered user on Medscape.org. If you are not registered on Medscape.org, please click on the "Register" link on the right hand side of the website to register. Only one answer is correct for each question. Once you successfully answer all post-test questions you will be able to view and/or print your certificate. For questions regarding the content of this activity, contact the accredited provider, CME@medscape.net. For technical assistance, contact CME@webmd.net. American Medical Association's Physician's Recognition Award (AMA PRA) credits are accepted in the US as evidence of participation in CME activities. For further information on this award, please refer to http://www.ama-assn.org/ama/pub/about-ama/awards/ama-physicians-recognition-award.page. The AMA has determined that physicians not licensed in the US who participate in this CME activity are eligible for **AMA PRA Category 1 Credits**™. Through agreements that the AMA has made with agencies in some countries, AMA PRA credit may be acceptable as evidence of participation in CME activities. If you are not licensed in the US, please complete the questions online, print the AMA PRA CME credit certificate and present it to your national medical association for review.

## Post-Test Questions

### Article Title: Using the National Death Index to Identify Duplicate Cancer Incident Cases in Florida and New York, 1996–2005

**CME Questions**

1. According to the registry data study by Wohler and colleagues, which of the following statements about death certificate-only (DCO) cases in the death state’s cancer registry identified as duplicates using the National Death Index (NDI), and potential duplicate cases, is ***correct***?
  - Of 5030 cases registered in both Florida and New York, approximately one quarter were DCO cases and three quarters were potential duplicates with the same diagnosis
  - Among Florida Cancer Data System (FCDS) and New York State Cancer Registry (NYSCR) patients who died and were registered in the other’s cancer registry, approximately 25% were registered with the same cancer diagnosis and roughly 50% had diagnosis dates within 1 year
  - Linkage with state death certificates only identified approximately three quarters of deaths among deceased cancer patients
  - Linkage with the NDI did not identify additional deaths among patients who died within the state where they were registered
2. According to the registry data study by Wohler and colleagues, which of the following statements about possible reasons why NDI identified deaths among state residents that were missed through routine death clearance is ***correct***?
  - Death clearance is usually performed quarterly
  - The registries and NDI both use probabilistic linkages for their matching algorithms
  - Because of data-exchange agreements limiting use of exchanged death certificate information, the FCDS missed data on almost 1% of deceased cancer patients who died out of state and whose death certificates were not shared with the FCDS
  - Death clearance is used to ascertain missing incident cases, with registry staff always using deaths from all causes
3. According to the registry data study by Wohler and colleagues, which of the following statements about potential strategies to avoid duplicate cases in state cancer registries would ***most*** likely be correct?
  - Standards from the North American Association of Central Cancer Registries for the reporting of high-quality cancer incidence data require that there be 1 or fewer duplicate cases per 500 cases reported
  - The registry initiating the NDI linkage should consider sharing relevant information with death state registries so that these registries can remove erroneous DCO cases from their databases
  - This study likely overestimated the number of potential duplicate cases between the FCDS and the NYSCR
  - This analysis included both living and deceased patients

**Evaluation**
